# Glucokinase (GCK) in diabetes: from molecular mechanisms to disease pathogenesis

**DOI:** 10.1186/s11658-024-00640-3

**Published:** 2024-09-08

**Authors:** Yasmin Abu Aqel, Aldana Alnesf, Idil I. Aigha, Zeyaul Islam, Prasanna R. Kolatkar, Adrian Teo, Essam M. Abdelalim

**Affiliations:** 1 Laboratory of Pluripotent Stem Cell Disease Modeling, Translational Medicine Division, Research Branch, Sidra Medicine, P.O. Box 26999, Doha, Qatar; 2 College of Health and Life Sciences, Hamad Bin Khalifa University (HBKU), Qatar Foundation, Education City, Doha, Qatar; 3 Diabetes Research Center, Qatar Biomedical Research Institute (QBRI), Hamad Bin Khalifa University (HBKU), Qatar Foundation (QF), PO Box 34110, Doha, Qatar; 4 Stem Cells and Diabetes Laboratory, Institute of Molecular and Cell Biology (IMCB), Agency for Science, Technology and Research (A*STAR), Proteos, Singapore, Singapore; 5 Department of Biochemistry and Department of Medicine, Yong Loo Lin School of Medicine, National University of Singapore, Singapore, Singapore; 6 Precision Medicine Translational Research Programme (PM TRP), Yong Loo Lin School of Medicine, National University of Singapore, Singapore, Singapore

**Keywords:** Glucokinase, Glucose, Insulin, Diabetes, Mutations, Stem cells, Beta cells, Pancreas, Liver

## Abstract

Glucokinase (GCK), a key enzyme in glucose metabolism, plays a central role in glucose sensing and insulin secretion in pancreatic β-cells, as well as glycogen synthesis in the liver. Mutations in the *GCK* gene have been associated with various monogenic diabetes (MD) disorders, including permanent neonatal diabetes mellitus (PNDM) and maturity-onset diabetes of the young (MODY), highlighting its importance in maintaining glucose homeostasis. Additionally, *GCK* gain-of-function mutations lead to a rare congenital form of hyperinsulinism known as hyperinsulinemic hypoglycemia (HH), characterized by increased enzymatic activity and increased glucose sensitivity in pancreatic β-cells. This review offers a comprehensive exploration of the critical role played by the *GCK* gene in diabetes development, shedding light on its expression patterns, regulatory mechanisms, and diverse forms of associated monogenic disorders. Structural and mechanistic insights into GCK’s involvement in glucose metabolism are discussed, emphasizing its significance in insulin secretion and glycogen synthesis. Animal models have provided valuable insights into the physiological consequences of *GCK* mutations, although challenges remain in accurately recapitulating human disease phenotypes. In addition, the potential of human pluripotent stem cell (hPSC) technology in overcoming current model limitations is discussed, offering a promising avenue for studying GCK-related diseases at the molecular level. Ultimately, a deeper understanding of GCK’s multifaceted role in glucose metabolism and its dysregulation in disease states holds implications for developing targeted therapeutic interventions for diabetes and related disorders.

## Introduction

Glucokinase (GCK) is a member of the hexokinase family, also known as hexokinase IV. It plays an essential role in glucose metabolism, crucial for sustaining blood glucose levels within normal ranges. It is a pivotal metabolic enzyme that catalyzes the first rate-limiting step of glycolysis in the pancreas and liver. GCK is responsible for catalyzing the ATP-dependent phosphorylation of glucose to glucose-6-phosphate (G6P), which triggers insulin secretion in pancreatic β-cells and glycogen synthesis in the liver [1]. GCK is also expressed in other tissues, including the intestine, hypothalamus, pituitary gland, lung, and spleen [2]. Unlike other hexokinases, GCK is characterized by an extremely low affinity for glucose and the lack of inhibition by its end-product, G6P. These characteristics mark GCK as the primary glucose sensor in many vertebrates, including humans [3].

GCK was first discovered as an enzyme in the rat liver and subsequently in the pancreas of obese mice during the 1960s [4, 5]. GCK has subsequently been the subject of intense studies due to its unique sigmoidal response to glucose. The strongest evidence of the GCK’s central role in glucose metabolism was provided in 1992 when a heterozygous *GCK*-inactivating mutation was reported to cause a mild type of monogenic diabetes (MD) termed maturity-onset diabetes of the young 2 (MODY2) [6]. In addition to MODY2, human genetic analysis established a link between GCK and other forms of MD, including the severe form of permanent neonatal diabetes mellitus (PNDM) caused by homozygous *GCK*-inactivating mutation(s) [7]. In contrast, mutations that increase GCK activity cause hyperinsulinism (GCK-HI), which is characterized by excessive insulin secretion [8]. Currently, more than 700 mutations in the *GCK* gene have been reported, distributed throughout its full length. Despite the high number of *GCK* mutations, around 80 in total have been characterized in vitro [9–12]. Moreover, the functional impact of the majority of GCK mutations remains unresolved.

This review provides an in-depth exploration of the pivotal role played by the *GCK* gene in the development of diabetes, shedding light on its expression patterns within pancreatic islets and the liver. Furthermore, it elucidates the intricate regulatory mechanisms governing GCK expression, which is crucial for understanding its contribution to diabetes pathogenesis. Furthermore, the article discusses the diverse forms of diabetes arising from GCK mutations and evaluates the potential of human and animal models in unraveling the underlying mechanisms of GCK-related diabetes development.

## Structure of GCK and mechanistic overview

The *GCK* gene is located on the short arm of human chromosome 7, specifically at the region (7p15.3-p15.1), and spans ten exons [9]. In mammals, a critical feature of the *GCK* gene is the presence of two distinct promoters separated by approximately ~ 30 kbp [1, 13]. The upstream promoter and its associated leader exon drive *GCK* expression in pancreatic islet cells and other non-hepatic tissues (neuroendocrine isoform). The downstream promoter and its associated leader promoter are active only in the liver [1]. These promoters, with their adjacent exons, specify the synthesis of 5ʹ-UTR of *GCK* mRNA and the first 15 amino acids of the GCK protein [1, 14]. Therefore, liver GCK protein differs from neuroendocrine GCK at their NH_2_ terminal ends. Despite this difference, liver and neuroendocrine GCK proteins have similar kinetic properties and are functionally indistinguishable [3, 14]. However, the regulation of *GCK* expression is distinct in the liver compared with endocrine cells at both transcriptional and posttranscriptional levels [14].

**Fig. 1:**
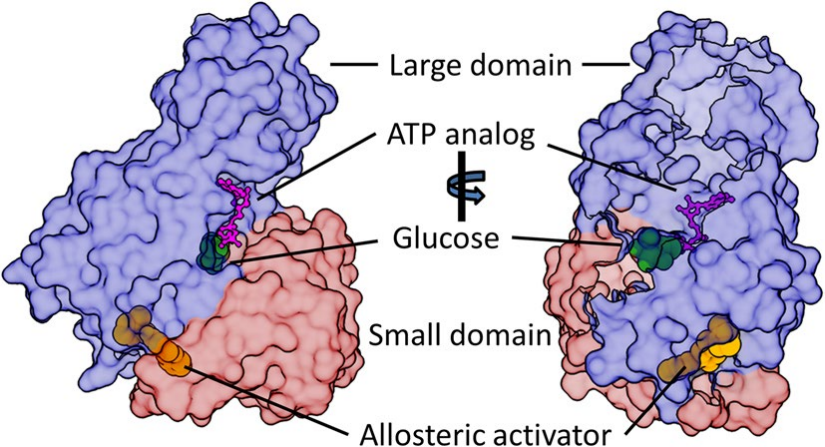
Surface representation of overall structure of glucokinase (GCK). The complex structure of glucose-bound GCK in the presence of the non-hydrolysable ATP analogue adenosine 50-(β,-γ-imino) triphosphate (AMP-PNP) and allosteric activator *N*-thiazol-2-yl-2-amino-4-fluoro5-(1-methylimidazol-2-yl)thiobenzamide (TAFMT) (PDBID: 3ID8). The complex comprises glucose (green, sphere), AMP-PNP (magenta, ball, and stick), and TAFMT (orange, sphere) with GCK in an active conformation. The conformational relationship of the large domain (light blue) and the small domain (salmon) exhibited a closed form

The unique structural characteristics of GCK allow it to act as a glucose sensor and as a crucial regulatory element in many metabolic processes [15]. GCK is a dynamic 52-kDa enzyme consisting of 465 amino acid residues, which fold into a large and a small domain [16]. Between the two domains is a cleft forming the active site where glucose binds. The orientation of the two domains is not static, as GCK exists in multiple conformational ensembles. Kamata and colleagues determined the crystal structure of human GCK using X-ray crystallography, providing explicit support for the model. Under two distinct crystallization conditions, GCK protein crystals were prepared with short truncations at the NH_2_-terminal end (11 or 15 amino acids), either in the presence of glucose and a ligand or without any ligand. The GCK protein generally comprises two globular domains (large and small) connected by three flexible loops forming a hinge. A narrow, deep cleft is formed between the two domains when glucose and the activator are present (Fig. 1). Both the glucose-binding pocket and the activator-binding site are located in the hinge region [15]. Because this GCK structure is very similar to the closed form of hexokinase I crystallized with glucose [17], it was thus identified as the closed form of the enzyme. Unlike GCK crystal structures determined with ligands, the super-open conformation is observed for the unbound GCK structure. As a result of two conformational changes compared with the closed form of GCK in this configuration, there is a much wider cleft space between the two domains of GCK due to the tilting and rotation of the small domain compared with the large domain (at an angle of 100°) in addition to extensive rearrangement of secondary structural elements within the small domain [15].

In the super-open conformation, GCK enzyme exhibits low glucose affinity and is catalytically inactive. During catalysis, glucose and ATP are bound at the active site in the closed conformation of GCK. The high-affinity form of the enzyme, free of substrates or products, is postulated to exist in an intermediate conformation between the super-open and closed states. As a result, this putative conformation is referred to as open [15]. GCK’s crystal structure in its open form still needs to be studied. However, an open form of hexokinase I can be modeled by X-ray diffraction studies of crystals formed in the absence of glucose [17]. Due to a slight twist of the small domain relative to the large domain, the open form displays a more comprehensive inter-domain cleft than the closed form. Compared with the complex molecular reorganization involved in the slow transition from super-open to open conformations, the transition from open to closed conformations would be extremely fast and easily reversible. The prediction of an intermediate open state for GCK has been supported by another study using targeted molecular dynamic simulations to determine when GCK transitions from a closed to a super-open state [15].

Several structures of GCK in complex with glucose and activators have been solved over the years [15, 18–23]. All the structures highlighted the same binding site for glucose and activators and validate that binding of activators does not cause conformational changes but help in stabilization of the active form of GCK. In fact, activators may help in shifting the glucose-dependent conformational equilibrium between open (inactive) GCK to closed (active) GCK as also indicated by kinetic studies [23]. These complex structures support the proposed theoretical model, where Asp105 acts as a base catalyst, Lys169 acts as an acid catalyst, and these help in the transfer of phosphoryl group between ATP and glucose during catalysis [22]. Contrarily, a slightly different model has been proposed on the basis of small-angle X-ray scattering (SAXS) in combination with crystallography studies [24]. The results suggest that GCK alternates between the active open and the active closed conformations to bind substrates and release products during the reaction cycle. These studies showed the existence of multiple conformations in solution, which may be required for efficient catalysis.

**Fig. 2:**
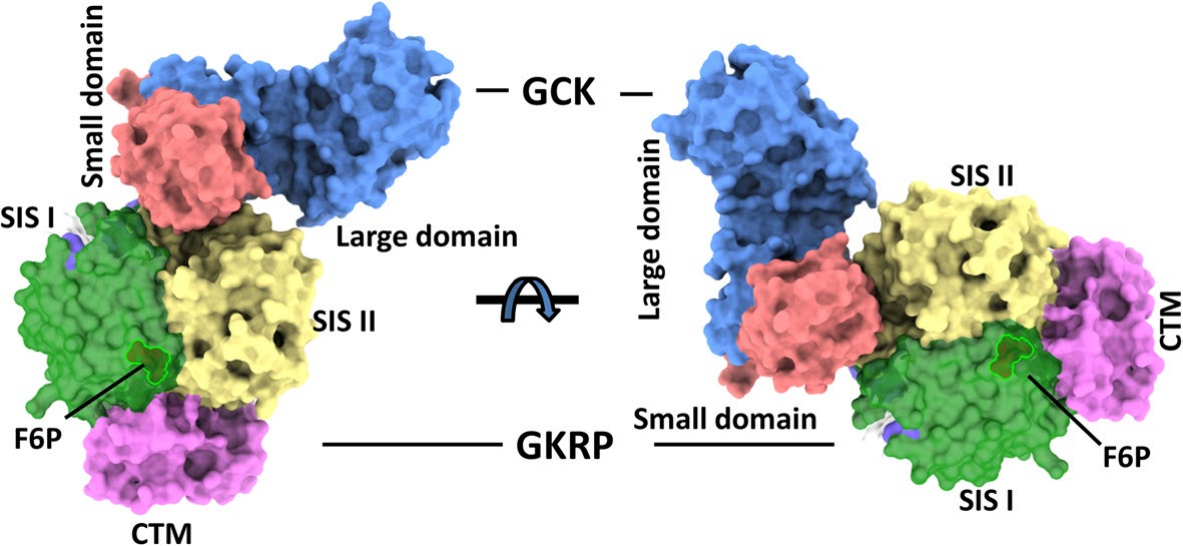
Surface representation of the overall structure of GCK/GKRP complex (PDBID: 4LC9). GCK is shown in light blue (large domain) and salmon (small domain). The structure of GKRP consists of two sugar isomerase (SIS) superfamily domains and a C-terminal extended all-helical motif (CTM). GKRP is depicted in green (SIS I), wheat (SIS II), and magenta (CTM). Fructose 6-phosphate (F6P) is shown as a sphere representation and binds at the interface of SIS (I and II) and CTM

On top of the conformational switch caused by substrate/ATP/activators, GCK is also regulated by GCK regulatory protein (GKRP), where GCK interacts with GKRP through the C-terminal domain. The crystal structure of *Xenopus laevis* GCK in complex with GKRP was solved to establish the molecular mechanism for the allosteric regulation of GCK by GKRP [25]. The structure revealed that GKRP binds GCK in a super-open conformation, interacts mainly through hydrophobic interaction, and modulates GCK activity by restricting a small domain of GCK [25]. Allosteric effectors of GKRP, such as fructose-1-phosphate (F1P) and fructose-6-phosphate (F6P), modulate the interaction between GCK and GKRP and indirectly modify the GCK activity [25–27]. F6P destabilizes the interactions (positive), while F1P stabilizes (negative) it, reciprocally affecting GCK-GKRP complex stability. Subsequently, the crystal structure of mammalian GCK-GKRP complex in the presence of F6P (Fig. 2) was solved, showing the molecular basis of regulation of GCK by GKRP [28]. The mammalian GCK-GKRP assembly structure reveals complex regulation by sugar phosphates similar to *Xenopus* GCK-GKRP, where GKRP has a distant sugar phosphate binding site from the GCK interface. This cascade regulation system plays a crucial role in blood glucose homeostasis, and the interface of the GCK-GKRP complex can be a promising drug target. GKRP plays a critical role in regulating GCK activity by sequestering GCK in the nucleus when glucose levels are low and releasing it into the cytoplasm when glucose levels rise. This interaction is crucial for glucose homeostasis [29, 30]. Furthermore, the C-terminal domain contains a nuclear localization signal (NLS) that is recognized by importins, which facilitate the nuclear import of GCK-GKRP complexes. This nuclear-cytoplasmic shuttling is also central to the regulation of GCK [29].

Overall, the unique structure of GCK is a crucial element of this enzyme that is intricately involved in glucose binding, regulation of enzymatic activity, and the overall control of blood glucose levels. Therefore, mutations in the *GCK* gene can either directly alter the enzyme’s substrate affinity or catalytic properties, leading to serious conditions such as PNDM, MODY2, and other forms of diabetes [31]. Understanding the structural and functional aspects of domains in GCK protein will be essential for unraveling the mechanisms behind glucose metabolism and diabetes-related disorders.

## GCK in pancreatic β-cells

Initially, GCK expression was identified in liver and pancreatic islet tissues [5, 32]. Subsequent studies have since detected GCK expression in multiple other tissues, including pancreatic acinar, brain, lung, kidney, and spleen [3]. Early findings in the human fetal pancreatic tissue revealed that GCK is expressed at weeks 17–19 of gestation as detected through cytosolic protein extracts [33]. Another study illustrated that GCK protein expression in the human fetal pancreas starts after week 15 of gestation [34]. Consistent with these findings, recent data generated using pancreatic cells derived in vitro from human pluripotent stem cells (hPSCs) revealed that GCK is expressed primarily in the mature stages of pancreatic cells [35, 36]. The expression of *GCK* mRNA has been demonstrated to peak significantly during pancreatic differentiation, persisting from day 18 until the completion of differentiation at day 29 [35].

**Fig. 3:**
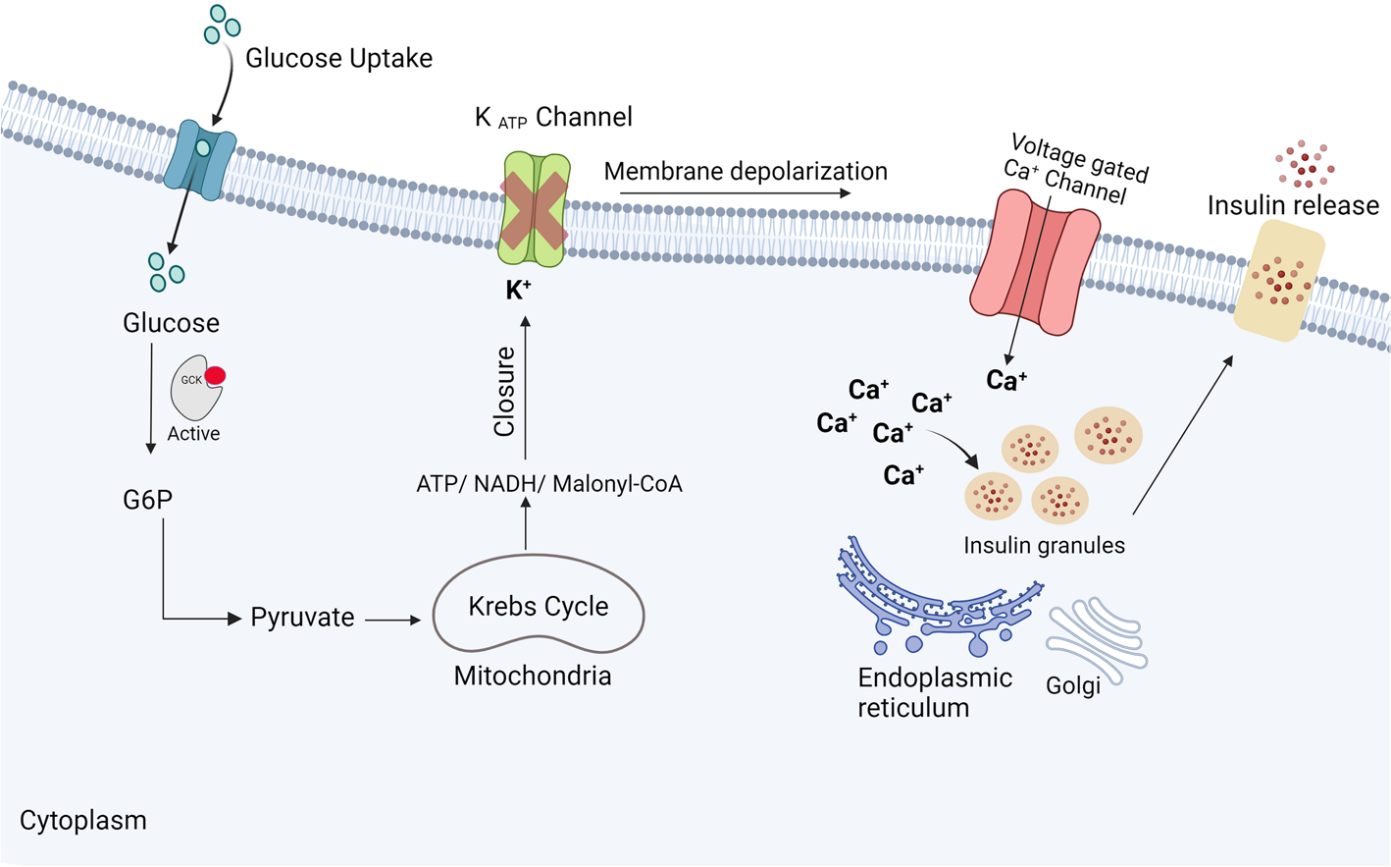
GCK role in pancreatic β-cells. Glucose enters pancreatic β-cells via low affinity glucose transporters. GCK then catalyzes the ATP-dependent phosphorylation of glucose into G6P. G6P starts the glycolysis and the Krebs cycle, which elevate the adenosine diphosphate (ADP) ATP/ADP ratio. Raised ATP/ADP levels result in K^+^ efflux, causing cell membrane depolarization and the opening of the voltage sensitive Ca^2+^ channels. The opening of Ca^2+^ channels elevate cytosolic Ca^2+^ levels, which, with other vital coupling factors, activate the endoplasmic reticulum and Golgi apparatus to secrete insulin granules from pancreatic β-cells

The function of GCK was first identified in pancreatic β-cells in the early 70s; the effect of glucose metabolism on the membrane electrical activity that resulted in secretion of insulin to maintain glucose homeostasis [37, 38]. GCK activity in human pancreatic islets was then described in 1985 [39, 40]. Importantly, it was disclosed that not all species have pancreatic β-cell GCK as it was found only in humans, rats, mice, and hamsters [40]. GCK in pancreatic β-cells is known to maintain glucose homeostasis by acting as a glucose sensor in the glucose metabolism pathway (Fig. 3) [4]. Subsequently, insulin is secreted from β-cells in response to high glucose levels. This acts as a key regulator of GCK activity in pancreatic β-cells [2, 41, 42], in which GCK is not inhibited by its end product, but is inhibited when the glucose level is back to normal [43]. The inflection point of the GCK-sigmoidal saturation curve with glucose is 4–8 mmol/L, which is very close to the threshold of insulin release (5 mM). As a result, when glucose level becomes close to the physiological threshold of insulin secretion, GCK activity reaches the plateau. This contributes to maintaining the fluctuating level of glucose in the blood [4, 44].

**Fig. 4:**
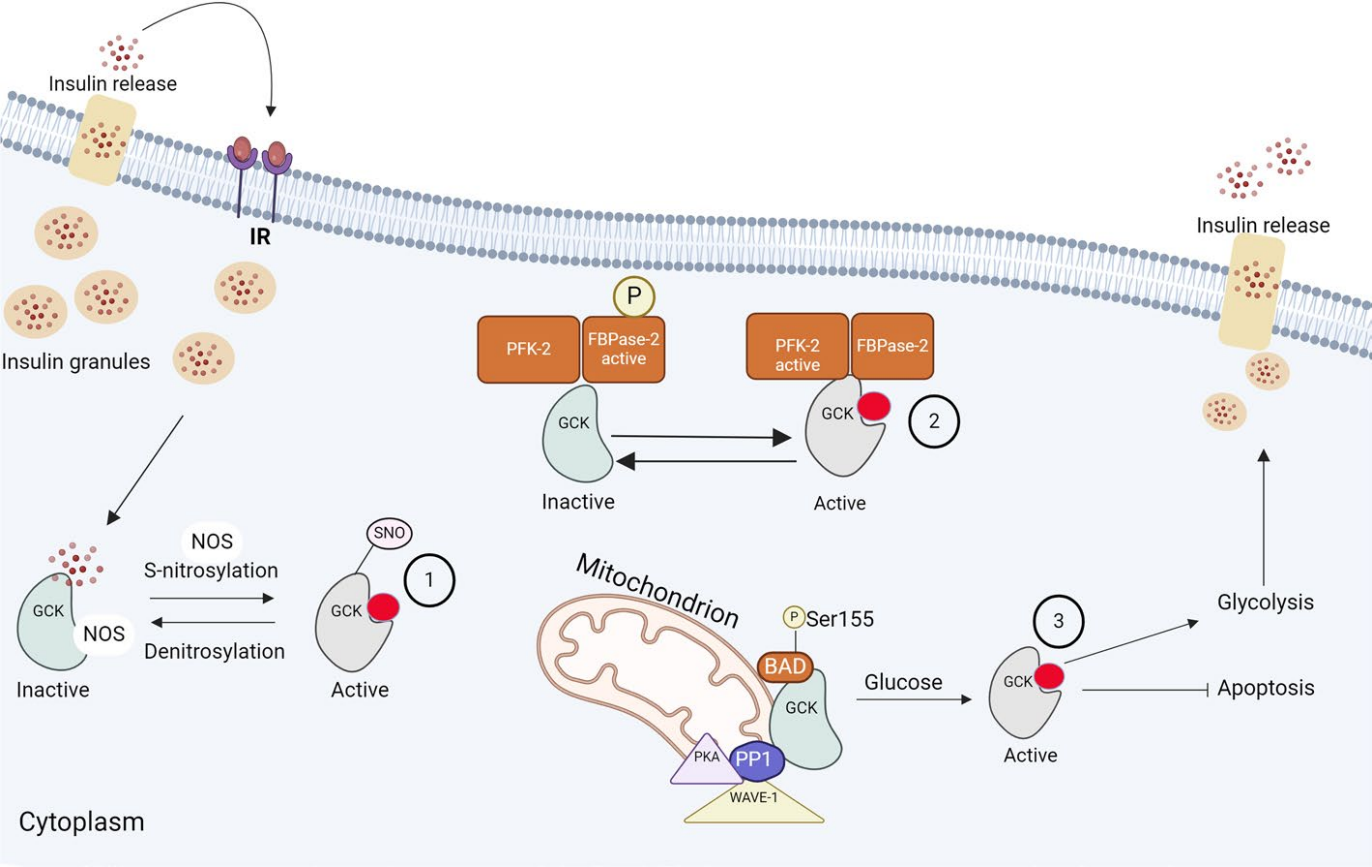
Regulation of GCK activity in pancreatic β-cells. In pancreatic β-cells, GCK activity is regulated by several binding partners: (**1**) GCK activity can be inhibited when it binds to insulin granules. The interaction between GCK and insulin granules is partly mediated by NOS. To reverse this interaction and stimulate GCK activity, NOS performs S-nitrosylation of GCK. (**2**) PFK-2/ FBPase-2. The regulation of GCK by PFK-2/ FBPase-2 involves direct binding of GCK and activation, depending on the phosphorylation status of FBPase-2. (**3**) Activation of GCK occurs via the BAD protein at the mitochondrial membrane. When phosphorylated at the BH3 domain, BAD binds to GCK near its active site, leading to GCK activation and subsequent insulin secretion. Furthermore, the interaction between BAD and GCK can provide protection against apoptosis

There are two different forms of GCK enzyme found in pancreatic β-cells: intrinsic high activity free-diffused form and low activity form that bind to some intracellular structures, mainly the mitochondria and insulin secretory granules [45, 46]. The binding affinity of GCK to the mitochondria may protect β-cells from apoptosis when they become intolerant to high glucose levels [47]. Therefore, this explains the importance of GCK function in minimizing the oligomerization process of Bax (the pro-apoptotic protein) and helps in the continuous release of cytochrome C from the mitochondria [47]. Separately, GCK activity is regulated and inhibited when GCK binds to insulin granules with the help of nitric oxide synthase (NOS) dimers that assist this regulation activity. However, this interaction is reversed after *S*-nitrosylation of the GCK enzyme, leading to the liberation of GCK from insulin granules (Fig. 4). This release of GCK will help in stimulating its activity as a post-transitional regulatory step in GCK-insulin granule interaction [8]. As a result, the localization and interaction of GCK with insulin granules are regulated by this physical interaction and chemical modulation. This GCK-bound form is a transient reservoir for its storage [8]. With the help of this association, the mobilization of cytoplasmic GCK in response to glucose change can be a rapid process when compared with the synthesis of new GCK molecules [8].

Phosphofructokinase-2/fructose bisphosphatase-2 (PFK-2/ FBPase-2) is a bifunctional regulatory enzyme that regulates glucose metabolism in pancreatic β-cells when bound to GCK (Fig. 4). This enhances GCK activity to regulate the intrinsic glucose level [48, 49]. Furthermore, the BH3 domain of BAD, a member of the BCL-2 pro-apoptotic protein family, binds to the GCK active site and regulates its activity [8]. BAD protein acts as a scaffold that binds and organizes different proteins, expediting the stabilization and activation of GCK and playing a crucial role in integrating the glycolysis and the apoptosis process in pancreatic β-cells through several proteins, including protein kinase A (PKA, cAMP-dependent protein kinase), protein phosphatase 1 (PP1, dual-specificity serine/threonine phosphatase), and Wiskott-Aldrich family member (WAVE1) [8]. GCK is mainly activated by the phosphorylation of BAD protein on Ser 155, resulting in stimulation of insulin release, which in turn improves the functionality and viability of β-cells [50, 51]. β-cell apoptosis, which results from inflammation or glucotoxicity, can therefore be prevented when glucose is metabolized by GCK [50, 51]. BAD also interacts with different pro-survival proteins that ultimately influence its proapoptotic function [50, 51]. The phosphorylation of BAD can result in the production of two residues, which are organized by intracellular glucose and insulin levels [8]. Importantly, it has been reported that the knockdown of BAD protein may result in a phenotype similar to that seen in the case of GCK knockdown, where it disturbs glucose-stimulated insulin secretion (GSIS) in β-cells [8].

Glucose is transported into pancreatic β-cells via low affinity glucose transporters such as glucose transporters GLUT1, GLUT2, and/or GLUT3 [2]. GLUT2 is known to be the main glucose transporter involved in glucose metabolism and is highly expressed in the liver and pancreatic β-cells in rodents [52], while GLUT1 and GLUT3 are recognized to be the main glucose transporters expressed in human islets [53]. Once glucose is transported into the cells, it gets phosphorylated into G6P with the help of the GCK enzyme. Subsequently, GCK expression reaches as high as five- to tenfold, independent of the glucose concentration [2]. Following phosphorylation, G6P undergoes glycolysis and enters the Krebs cycle, leading to increased oxidative phosphorylation in the mitochondria that generates more ATP in the cell [2]. The elevated ATP/ADP ratio in the cytosol is one of the most important coupling factors controlling insulin release [54]. When the ratio of ATP/ADP rises inside cells, K^+^ efflux is diminished [54]. As a result, the SUR-1/Kir6.2 K_ATP_ channel complex is inhibited, causing a depolarization of the cell membrane, and opening of the voltage sensitive Ca^2+^ channels. This results in an elevation of the cytosolic Ca^2+^ level [2]. Subsequently, insulin-containing granules will be secreted from β-cells [55]. Insulin is secreted in a biphasic manner; the first phase occurs after a few minutes of glucose stimulation, followed by a reduction of insulin release. After a few minutes of the first phase, the second phase begins, and 30–40 min after glucose stimulation, insulin secretion reaches a peak [56].

β-cell proliferation can be regulated in the presence of glucose through inducing β-cell glycolytic flux [57]. GCK expression increases when glucose levels increase, resulting in IRS-2 and cyclin D2 stimulation and upregulation, where these genes are involved in β-cell proliferation [57, 58]. IRS-2 is known to be essential in keeping the β-cell mass maintained and preventing β-cells from going under the apoptotic mechanism, which has a crucial role in preventing the occurrence of diabetes [59, 60]. Therefore, upregulation of IRS-2 via GCK activation could improve β-cell proliferation [60]. It has been shown that mice with heterozygous mutation in GCK β-cell (βGck^+/−^) showed an increase in ER stress and β-cell apoptosis when fed with a diet rich with linoleic acid and sucrose compared with wild type mice [61]. The use of GCK activator (GKA) increases the expression of IRS-2 and reduces CHOP and BAX protein expression, which trigger ER stress and β-cell damage. Reducing the expression of those proteins can lead to recovery of the apoptotic effect of ER stress in β-cells in prediabetic or early stages of diabetes where the β-cell damage is not massive [62, 63]. Similarly, the ubiquitin–proteasome system (UPS)—a type of posttranslational modification system—helps in regulating the activity of GCK in pancreatic β-cells [64, 65]. The UPS is involved during the synthesis and secretion of insulin after glucose induction, as well as the expression and function of ATP-sensitive K^+^ channels, which have an important role in maintaining glucose homeostasis in the body [64, 65]. Small ubiquitin-like modifier protein 1 (SUMO-1) is a protein that binds to GCK and contributes to the regulation of its activity by increasing its activity and stability [64, 65]. While it has been described that SUMO-conjugation binds to the closed conformational form of GCK, it is not clearly understood whether this binding causes the closed conformation form, or whether it is because of the SUMO-GCK interaction. This type of GCK regulation is considered a posttransitional modification process that contributes to the regulation of GCK function [64, 65].

At the transcriptional level, Pdx1 can regulate the transcriptional activity of the *Gck* promoter in the pancreatic β-cells [66]. PDX1 transcription factor is a primary regulator of pancreatic development and the differentiation of progenitors into pancreatic β-cells [67]. PDX1 regulates the expression of some islet-specific genes such as *GCK* [66]. A study using Chinese hamster ovary (CHO) cells showed that PDX1 expression can increase the reporter activity of the *GCK* promoter in pancreatic β-cells via binding on the upstream promoter element 3 (UPE3) in the *GCK* promoter [67]. Nevertheless, it has been shown that specific disruption of the *Pdx1* gene did not influence GCK expression [68, 69].

## GCK in other pancreatic islet cells

Besides the liver and pancreatic β-cells, GCK is also present in other pancreatic islet cells, such as pancreatic α- and δ-cells [70]. The endocrine part of the pancreas responds to changes in blood glucose levels by secreting hormones, either glucagon from α-cells in a fasting state or insulin from β-cells after or during meals [71, 72]. The liver responds to glucagon secretion by mobilizing glucose from its intracellular glycogen storage, while the secretion of insulin results in increased uptake of glucose from the portal vein [72]. Therefore, this system is regulated by glucose and has positive feedback on insulin secretion [71] but negative feedback on glucagon secretion [73].

In α-cells, glucose requires GCK to suppress the expression of glucagon that is released to prevent hypoglycemia, especially during hyperglycemia. The general properties of GCK in α-cells are similar to that of GCK in β-cells in terms of saturation (high S_0.5_) and non-inhibition property by its end product G6P [74]. Although the glycolysis process in α-cells and β-cells is similar, the ATP produced is less in α-cells because the oxidative phosphorylation efficiency resulting from high expression of uncoupling protein 2 is lower [75, 76]. The knockdown of GCK affects the regulation of glucagon secretion [77], as is evidenced from mice with inactivated GCK in α-cells [78]. Due to the absence of GLUT2 transporter in pancreatic α-cells, intracellular glucose level is maintained between 1/2 and 2/3 of serum level. This range is sufficient for GCK to serve as a glucose sensor in these cells [79].

The sorting of pancreatic α-cells from rat islets revealed that GCK stands as the sole hexokinase enzyme detected within the determined limit [77]. This finding underscores the minimal expression of hexokinase I, as validated in earlier studies on α-cells [78]. The presence of GCK in α-cells has also been noticed in enriched islets of rats treated with streptozotocin [70]. Moreover, abnormalities in GCK gene expression have been noted to disrupt insulin release triggered by glucose induction and impair the release of glucagon through glucose suppression [80]. This particular defect has been observed in certain instances of MODY2 within families harboring congenital mutations in the GCK gene [81].

Studies conducted on MODY2 patients with *GCK* heterozygous mutation showed increased glucagon secretion from α-cells during the hyperinsulinemic/hypoglycemic clamps, which are stimulated at high glucose concentrations. However, the reason is unclear: is it because of the direct consequence of GCK defects in α-cells, or is it influenced by glucose-sensing neurons in the central nervous system [82]. To elucidate the critical role of GCK in α-cell glucose sensing, mice with α-cell-specific *Gck* deletion have been generated. Published data underscore the importance of the intrinsic regulation of α-cells [78]. Inactivation of *Gck* in α-cells disrupt the ATP/ADP ratio and the K_ATP_ channel closure in α-cells, leading to hyperglucagonemia in the fed state and an increment in glucose produced by the liver [78]. Furthermore, glucagon secretion is suppressed in *Gck*-deficient α-cells, even in the presence of other extrinsic regulatory factors such as paracrine, hormonal, and neuronal control. This collective evidence underscores the importance of intrinsic regulation of GCK within the α-cell for glucagon secretion [78]. Moreover, abnormal suppression of glucagon will affect hepatic glucose metabolism, resulting in the induction of prediabetic conditions [78].

In addition, it has been reported that the pancreatic polypeptide cells (PP) cells do express GCK at a low level, while other hexokinases are not expressed [77].

## GCK in liver

The regulation of blood glucose levels by the liver is more complex than that of pancreatic islets. By converting glucose to G6P, GCK enhances glycogen synthesis and contributes to removing glucose from the portal vein [83]. The liver also plays an essential role in endogenous glucose production to maintain normal glucose levels during fasting [84]. Therefore, GCK activity is required for efficient glucose clearance after a meal, whereas it should be inactive during fasting to prevent glucose conversion to G6P [14, 84]. In the liver, GCK expression is strictly driven by the presence of insulin. It is abolished in the liver of insulin-deficient rats [1, 85]. The induction of GCK expression by insulin has been shown to occur at the transcriptional level, where insulin triggers around a 15-to-30-fold increase in *Gck* mRNA independently of glucose [86]. In addition to the transcriptional induction of *GCK* gene expression, GCK is also subject to several protein–protein interactions that produce a wide range of physiological consequences. The liver’s earliest and best-characterized partner of GCK is glucokinase regulatory protein (GKRP) [87, 88]. GKRP is a 65 kDa monomeric enzyme primarily localized in the liver nucleus [83, 89]. GKRP has also been detected in the cytosol and mitochondria complex with GCK [90]. Since GKRP was discovered more than 30 years ago, it has been extensively studied using various biochemical, biophysical, and structural methods. Structural analysis revealed that GKRP binds the super-open conformation of GCK mainly through a hydrophobic interaction [25]. This binding makes GKRP a competitive inhibitor of glucose association with the enzyme [25]. Furthermore, GKRP binding to GCK sequesters the complex in the nucleus of hepatocytes [89]. The exact mechanism by which GKRP mediates GCK nuclear translocation remains unclear. A possible mechanism is via a signaling sequence. Importantly, this mechanism was identified in the pancreatic GCK in a region of the enzyme that is also conserved in the liver isoform [91].

GKRP-mediated GCK regulation is subject to further modulations. For example, phosphorylated carbohydrates such as fructose-6-phosphate (F6P) enhance the interaction between GKRP and GCK [92]. The analysis of GKRP association with a fluorescent variant of human GCK revealed that GKRP binding to GCK is a two-step process. The first step is forming an initial encounter complex, while the second step is the conformational equilibrium between two GKRP-GCK states [93]. This analysis described that F6P enhances GKRP-GCK interaction by promoting the formation of the initial encounter complex. Another modulator of GKRP is fructose 1-phosphate (F1P) [25]. In contrast to F6P, F1P weakens GKRP-GCK interaction by altering the hydrophobic interaction between GCK and GKRP [25].

Posttranslational (PT) processes have also been shown to modulate liver GKRP–GCK interaction. For example, GCK SUMOylation stabilizes and activates GCK [8, 91]. However, SUMOylation of GCK impairs GKRP’s capacity to promote the nuclear translocation of GCK [8, 91]. Moreover, the attachment of one or more SUMO proteins to GCK at Lys12, Lys13, Lys15, and Lys346 may conceal the NES (nuclear export signal), preventing the nuclear export of GCK while it is SUMOylated [8, 91]. Another PT event is the acetylation of GKRP near the N-terminus by P300 acetyltransferase. This prolongs GKRP lifespan and increases its inhibitory effects [94]. The presence of several mechanisms regulating GKRP–GCK interaction highlights the physiological importance of regulating GCK activity in the liver.

## Genetic variants of *GCK* in monogenic diabetes

Mutations in the *GCK* gene can result in various forms of diabetes. These mutations can either directly alter the enzyme’s substrate affinity or catalytic properties, leading to severe conditions such as MODY2 and other forms of diabetes [31]. More than 600 *GCK* mutations have been reported to cause different monogenic glycemic disorders (Table 1) [9]. Therefore, studying the defects in GCK expression in the liver, pancreas, or both has a vital role in understanding the cause of monogenic diabetes [95]. Several studies explained how the reduction in the activity of GCK in pancreatic β-cells in transgenic mice resulted in insulin secretion reduction in response to glucose while showing no significant change in fasting plasma glucose level or glucose tolerance [1, 96]. Those studies could confirm the contributive role of global GCK defect (pancreatic and hepatic GCK) in developing MODY2 disease [1, 96]. There are three types of mutations identified in the *GCK* gene: (i) missense mutations that affect the sequence of the *GCK* gene and prevent the normal conformational changes; (ii) nonsense mutations that result in the generation of truncated molecular form of GCK; and (iii) deletion and splicing mutation(s) that may result in the synthesis of defective mRNA that cannot translate to normal and functional protein [97].

**Table 1:** Examples of the most common mutations in the *GCK* gene

	Mutation point	Region	Protein change	References
MODY2				
1	c.-71G>C	Islet promoter	NA	[121]
2	c.106C>T	Exon 2	p.Arg36Trp	[136]
3	c.157G>T	Exon 2	p.Ala53Ser	[130, 136]
4	c.175C>T	Exon 2	p.Pro59Ser	[137]
5	c.182A>G	Exon 2	p.Try61Cys	
6	c.184G>A	Exon 2	p.Val62Met	[133, 134]
7	c.208G>A	Exon 2	p.Glu70Lys	[130, 132, 138]
8	c.214G>A	Exon 3	p.Gly72Arg	[133, 139]
9	c.234C>G	Exon 3	p.Asp78Glu	[137]
10	c.239G>C	Exon 3	p.Gly80Ala	[130, 136]
11	c.260T>C	Exon 3	p.Val86Ala	[140]
12	c.349G>A	Exon 3	p.Gly117Ser	
13	Deletion in the 5ʹ splice site of intron 4	intron 4	NA	[120]
14	Deletion of the T of the GT in the splice donor site of intron 4 and the following 14 base pairs	Intron and exon 4	K161 + 2de115	[98]
15	c.401C>T	Exon 4	p.Leu134Pro	[141]
16	c.410A>G	Exon 4	p.His137Arg	[130]
17	c.413A>C	Exon 4	p.Gln138Pro	[141]
18	c.437T>G	Exon 4	p.Leu146Arg	[139]
19	c.451_453delTCC	Exon 4	p.Ser151del	[142]
20	c.457 C>T	Exon 4	p.Pro153Ser	[141]
21	c.469G>A	Exon 4	p.Glu157Lys	
22	c.475A>G	Exon 4	p.Ile159Val	[140]
23	c.478G>C	Exon 4	p.Asp160His	
24	c.480_482dupTAA	Exon 4	p.Asp160_Lys161 insAsn	[109]
25	c.493C>T	Exon 5	p.Leu165Phe	[143]
26	c.502A>C	Exon 5	p.Thr168Pro	[130, 136]
27	c.505 A>G	Exon 5	p.Lys169Glu	[141]
28	c.512T>C	Exon 5	p.Phe171Ser	[140]
29	c.524G>A	Exon 5	p.Gly175Glu	[130]
30	c.544G>T	Exon 5	p.Val182Leu	[144]
31	c.544G>A	Exon 5	p.Val182Met	[145]
32	c.544G>A	Exon 5	Val182Met	[98, 145]
33	c.556C>T	Exon 5	Arg186 to stop	
34	c.562G>A	Exon 5	p.Ala188Thr	[101]
35	c.579 G>T	Exon 5	p.Gly193Gly	[141]
36	c. 595 G>A	Exon 6	p.Val199Met	
37	c.608T>C	Exon 6	p.Val203Ala	[98, 130, 132, 145]
38	c.617C>T	Exon 6	p.Thr206Met	[143]
39	c.622G>A	Exon 6	p.Ala208Thr	[139]
40	c.626C>T	Exon 6	p.Thr209Met	[136]
41	c.629T>C	Exon 6	p.Met210Thr	[130]
42	c.637T>C	Exon 6	p.Cys213Arg	[130, 136, 146]
43	c.676G>A	Exon 6	p.Val226Met	[130, 136]
44	c.697T>C	Exon 6	p.Cys233Arg	[144]
45	c.703A>G	Exon 6	p.Met235Val	[109]
46	c.755G>A	Exon 6	p.Cys252Tyr	[146]
47	c.766G4A	Exon 6	p.Glu256lys	[98]
48	c.769T>C	Exon 6	p.Trp257Arg	[101]
49	c.781G>A	Exon 6	Gly261Arg	[98, 100, 130]
50	c.787T>C	Exon 6	p.Ser263Pro	[139]
51	c.793G>A	Exon 6	p.Glu265Lys	[143, 144]
52	c.835G>C	Exon 6	p.Glu279Gln	[98]
53	c.713T>C	Exon 7	p.Met238Thr	[140]
54	c.819T>G	Exon 7	P.Tyr273X	
55	c.841T>G	Exon 7	p.Ser281Ala	
56	c.895G>C	Exon 8	Gly299Arg	[98, 147]
57	c.898G>C	Exon 8	Glu300Gln	[98]
58	c.898G>A	Exon 8	p.Glu300Lys	[98, 130, 132]
59	c.922A>T	Exon 8	p.Arg308Trp	[109]
60	c.926T>C	Exon 8	p.Leu309Pro	[98, 130]
61	c.944T>C	Exon 8	p.Leu315Pro	[141]
62	c.950A>C	Exon 8	p.His317Pro	[140]
63	c.1007C>T	Exon 8	p.Ser336Leu	[130, 148]
64	c.1016A>G	Exon 8	p.Glu339Gly	[139]
65	c.1055T>C	Exon 8	p.Leu352Pro	[140]
66	Splicing mutation: mutation of the splice acceptor site in intron9 from AG to AC	Intron 9 and exon 10	S418-1G to C	[145]
67	c.1030G>T	Exon 9	p.Asp344Tyr	[149]
68	c.1099G>A	Exon 9	p.Val367Met	[130, 136]
69	c.1121_1132del12	Exon 9	p.Val374_Ala377del	[142]
70	c.1129C>T	Exon 9	p.Arg377Cys	[139]
71	c.1136C>T	Exon 9	p.Ala379Val	[144]
72	c.1229G>T	Exon 9	p.Gly410Va	[141]
73	c.1240A>G	Exon 9	p.Lys414Glu	[128, 130]
74	c.1222G>T	Exon 10	p.Val408Leu	[140]
75	c.1256T>G	Exon 10	p.Phe419Cys	
76	c.1258A>G	Exon 10	p.Lys420Glu	[144]
77	c.1322 C>T	Exon 10	p.Ser441Leu	[141]
78	c.1358C>T	Exon 10	p.Ser453Leu	[139]
MODY2-PNDM				
79	c.437T>C	Exon 4	p.Leu146Pro	[150]
80	c.502A>G	Exon 5	p.Thr168Ala	[151]
81	c.629T>A	Exon 6	p.Met210Lys	[139]
82	c.790G>A	Exon 6	p.Gly264Ser	[107, 139]
83	c.1133C>T	Exon 9	p. Ala378Val	[107]
84	c.1190G>T	Exon 9	p.Arg397Leu	[109]
HH				
85	c.191C>A	Exon 2	p.Ser64Tyr	[152]
86	c.194C>T	Exon 2	p.Thr65Ile	[125, 135]
87	c.203G>T	Exon 2	p.Gly68Val	[153]
88	c.295T>A	Exon 3	p.Trp99Arg	[125, 135, 146]
89	c.296G>T	Exon 3	p.Trp99Leu	[123]
90	c.591G>T	Exon 6	p.Met197Ile	[123]
91	c.641A>G	Exon 6	p.Tyr214Cys	[129, 135, 154]
92	c.1361_1363dupCGG	Exon 10	p.Ala454dup	[123]
93	c.1363C>A	Exon 10	p.Val455Met	[124, 130, 132]
94	c.1367C>T	Exon 10	p.Ala456Val	[126, 128, 129, 135]

*GCK* mutations have been identified across exons 2–10, with a notable concentration observed in exons 5–8 [98]. Exon 5 is particularly significant as it encodes the region responsible for inducing the enzyme’s conformational change upon glucose binding, leading to the closure of the active site cleft [99]. Exons 6–8 encode residues crucial for both the active site and its cleft [100, 101]. Therefore, research efforts have focused on studying mutations within these exons [102, 103] and some investigations into mutations reported in exon 1 [103]. Routine sequencing checks have also uncovered numerous cases of polymorphisms in the *GCK* gene, with the IVS9+8T>C variant being the most commonly identified polymorphism [103].

### PNDM

PNDM, a rare and severe form of monogenic diabetes, typically emerges within the first 6 months of life [104]. Generally, these patients have first-degree relatives with reported glucose intolerance. Patients with PNDM require a lifelong insulin treatment supply that is usually accompanied by sulfonylurea [105]. This condition arises from autosomal recessive mutations, either due to homozygous mutations or compound heterozygosity involving nonsense, frameshift, or missense mutations, leading to complete loss of GCK function [7, 106, 107]. The first discovery of PNDM caused by *GCK* mutations was documented in 2001, with two reported cases from Norway and Italy [7]. An example of homozygous inactivating mutation causing PNDM is Ala378Val (A378V). This missense mutation, located near the GCK active site, disrupts the binding ability of GCK with glucose [107]. There are other types of homozygous mutations that present in splice donor sites and can result in forming an inactive GCK protein [107]. PNDM can also result from compound heterozygous mutations, such as (G264S/IVS8+2T G), where one allele has a mutation in the splice site in one exon (IVS8+2T→G) and the other allele has a missense mutation such as Gly264Ser [107]. Although the Gly264Ser missense mutation exhibits normal enzymatic activity, the exact cause of the disease is not fully understood. Nonetheless, severe PNDM has been observed in patients harboring such mutations [107]. Another rare example of homozygous mutation within the *GCK* gene linked to PNDM is the Gly223Ser mutation. This mutation occurs within the β-sheet, particularly the hydrophobic core in the large domain of GCK [108]. In this mutation, there is a substitution of glycine to serine, which affects the activity and structure of GCK [109]. Consequently, patients with this mutation develop severe hyperglycemia and ketoacidosis symptoms due to the complete loss of GCK, aligning with the PNDM phenotype [108]. Up to date, most PNDM due to *GCK* homozygous mutations has been diagnosed during infancy. However, there have been some rare cases of *GCK* homozygous mutations diagnosed outside infancy. For example, Raimondo et al. reported the first two cases of PNDM diagnosed at 9 and 15 years of age, with mutations c.478G>A (p.D160N) and c.676G>A, (p.V226M), respectively [110].

### MODY2

Heterozygous mutation in the *GCK* gene is known to cause MODY2, characterized by mild fasting hyperglycemia. In MODY2, *GCK* gene shows functional defects reported in approximately 1 out of 1000 of the population [111]. The clinical similarities between MODY2 and other diabetes types can complicate diagnosis, often resulting in misdiagnosis and inappropriate treatment with insulin or oral hypoglycemic agents. Patients typically do not require pharmacological intervention, and their risk of diabetes-related vascular complications is relatively low [111–113]. The discovery of *GCK* mutations as the genetic cause of MODY2 first occurred in 1992 groups [6, 114]. Compared with individuals diagnosed with transcription factor MODY who develop diabetes during young adulthood or adolescence and advance to marked hyperglycemia, accompanied by escalating treatment needs and an increased risk of diabetes-related complications, MODY2 only results in mild hyperglycemia. This unique pathophysiology observed in individuals with MODY2 emphasize the importance of considering them as a separate genetic subgroup, markedly divergent from other MODY subtypes [115].

Heterozygous mutations in *GCK* present in two forms: loss-of-function (MODY2) or gain-of-function (GCK-hyperinsulinaemic hypoglycemia, GCK-HH). Loss-of-function mutations in GCK lead to defective glucose sensing mechanism in pancreatic β-cells, resulting in reduced insulin secretion due to an increased glucose threshold needed for insulin release. Consequently, this leads to elevated fasting glucose levels and the development of MODY2 diabetes [111, 116]. Furthermore, patients with MODY2 exhibit decreased glycogen production in the liver in response to reduced insulin release, along with increased hepatic glucose production (gluconeogenesis) post-meals [117]. This contributes to the hyperglycemic conditions in MODY2, supported by studies that showed reduced glucose cycling in the liver and abnormally high endogenous glucose production relative to plasma glucose concentration in affected individuals [117, 118]. Notably, elevated fasting glucose levels in MODY2 do not correlate with body mass index (BMI), as this is observed to a similar degree in slim and obese people [119].

Some mutations in the intronic region of the *GCK* gene can cause MODY2 with hyperglycemia symptoms. These mutations can result in the deletion of entire exons or the formation of abnormal transcripts. For example, the deletion of 15 base pairs in the donor splice site of intron 4, where the “T” of the “GT” is removed with the subsequent 14 base pairs, leads to the formation of two defective transcripts; one transcript lacks the entire exon 5, while the other is missing the last eight codons of exon 4 [120]. Deletion mutations in the intronic region, particularly in the donor splice site, have been demonstrated to cause a severe form of glucose intolerance compared with the form resulting from the *GCK* point mutations, as they have a more detrimental effect on insulin secretion [120].

Mutations in promoter regions of important β-cell genes can also cause the phenotype observed in certain cases of MODY2, even in the absence of mutations within the exons or introns of the *GCK* gene [121]. Studies have revealed mutations such as −71G>C, which are detected during sequencing of the *GCK* promoter region. This mutation occurs in a non-conserved region of the human *GCK* promoter, leading to a defect in promotor activity of up to fourfold [121]. This finding underscores the vital role of non-coding regions in developing diseases such as MODY2 and emphasizes the necessity of including these regions in disease screening protocols [121].

### GCK-HH

GCK hyperinsulinism (GCK-HI) constitutes a rare congenital form of HI. Mutations associated with GCK-HI induce an increase in GCK enzymatic activity, leading to a lowered glucose threshold for glucose-stimulated insulin secretion (GSIS) in pancreatic β-cells [122]. In 1998, hyperinsulinaemic hypoglycemia due to heterozygous gain-of-function mutation (GCK-HH) was identified [111, 123, 124]. This mutation increases the affinity of GCK for glucose, resulting in a lower threshold for insulin release [42]. Consequently, previous studies reported symptoms of stable and persistent hypoglycemia correlated with increased insulin release. This increased insulin secretion is observed in response to intravenous stimulation of glucose, resulting in hyperinsulinemia [42]. The spectrum of hyperinsulinism ranges from mild to intermediate to severe forms. Treatment approaches depend on the severity of each case; for example, diazoxide is usually used for intermediate cases, while more severe instances may necessitate pancreatectomy to manage the hypoglycemic condition [123]. In addition, it has been reported that there are over 11 activating GCK mutations, with the majority located in the allosteric region of GCK where the GCK activators bind [125, 126].

It has been reported that the Val455Met mutation, located in exon 10 of the *GCK* gene, is a conservative missense mutation linked to GCK-HH, despite the fact that this mutation is not present in the GCK–glucose binding site and there being no familiar missense mutations associated with MODY2 in that region [124]. Hence, many kinetic analyses have been performed to elucidate this association. Interestingly, analyses demonstrated that the Val455Met mutation enhances the affinity of GCK for glucose, resulting in an increased glycolysis rate at low concentrations of glucose and elevated insulin secretion at lower plasma concentrations. This could explain the hypoglycemia and hyperinsulinemia observed in families carrying this mutation [124].

Most GCK-HH mutations occur within the enzyme’s active site, impacting its catalytic activity through disturbing its binding with glucose [127]. Furthermore, other mutations located in different sites, such as promoter regions, may influence the expression of tissue-specific GCK in hepatic or pancreatic β-cell splicing sites, GCK regulatory domains such as the fructose 6-/fructose 1-phosphate regulatory protein binding site, and GCK regulatory sites [127]. A study used mice with specific genetic backgrounds to understand the association between missense mutations in GCK and glycemic disease [128]. These mice have been engineered to carry either activating mutations, such as Ala456Val [129], or inactivating mutations, such as Lys414Glu [130], in the *Gck* gene. Results revealed that mice heterozygous for the inactivating mutation (Lys414Glu) displayed hyperglycemia, representing the phenotype of MODY2, while mice with the heterozygous activating mutation (Ala456Val) showed hypoglycemia, characteristic of GCK-HH [128]. Consequently, the threshold for GSIS from pancreatic β-cells has been changed depending on the mutation type, either activating or inactivating; mice with activating mutations showed elevated plasma insulin concentrations, whereas those with inactivating mutations exhibited slightly lower levels [128]. Nevertheless, some reported mutations, such as S263P and G264S, may be catalytically normal, but if overexpressed in certain cells such as HEK293 cells and MIN6 β-cells, they will lead to the generation of misfolded proteins that result in destabilization and/or cellular dimerization/aggregation with an accelerated rate of degradation [131].

Moreover, hepatic GCK activity has been found to be affected in mice with activating mutation, resulting in a significant downregulation of GCK protein levels in the liver [128]. Such observation could be explained by the mutation causing the translation of the unstable GCK protein [132]. Another explanation is that there may be disruption in the interaction between GCK and its regulatory protein (GKRP) [128].

Taken together, mutations in *GCK* causing MODY2 not only affect the kinetics of the enzyme, but also impair its stability [131, 132], its interaction with other regulatory proteins such as GKRP [133, 134], and its ability to bind with other bifunctional enzymes such as PFK-2/FBPase-2 [133], or binding with some activators in the allosteric site [134]. Furthermore, mutations causing GCK-HH to impair various enzymatic mechanisms, including increasing the GCK–glucose binding affinity and other mutations, help to change the GCK form into its active form [135].

## *GCK* variants linked to type 2 diabetes (T2D)

It has been reported that a high proportion of patients with T2D carry some rare *GCK* variants. Depending on the type of *GCK* variant, these patients can have a glycemic phenotype and a treatment response consistent with known GCK monogenic diabetes [155]. For example, patients with T2D with known pathogenic *GCK* variants such as c.214G>A and c.659G> often display lower fasting glucose and C-peptide levels similar to MODY2. In contrast, those with benign variants such as c.1024A>C typically exhibit distinct glycemic phenotypes characterized by higher fasting glucose and c-peptide levels T2D [156]. Moreover, it has been investigated that some common variant in the *GCK* gene with a minor allele frequency of ≥ 0.01 is linked with T2D and some metabolic traits. A novel 3′ untranslated region (3′UTR) SNP, in chr7:44184184-G/A, has been found to be associated with the post-absorptive carbohydrate oxidation rate and during a hyperinsulinemic–euglycemic clamp, as well as its association with T2D. Such variants in the *GCK* gene could influence the carbohydrate oxidation rate and are reported to be associated with T2D [156]. The *GCK* rs1799884 variant has been identified as another example of a *GCK* variant associated with T2D, specifically in Caucasians [157] and Malaysians [158]. This association has been established by studying the genotypic and allelic frequencies of this variant in combination with variants in *GCKR* (rs780094), and *G6PC2* (rs560887), revealing significant differences in patients with T2D and controls and highlighting the combined impact of these variants on T2D risk [158]. The presence of these alleles results in an elevation in the insulin secretion set point, disrupting physiological glucose homeostasis and resulting in increased FPG levels according to the number of available risk alleles, thereby increasing susceptibility to T2D [159].

Furthermore, another study showed a negative association between FPG and expression of GCK in patients with T2D, which indicates that activation of GCK could be essential for β-cell adaptation and proper glycemic control. Those results are consistent with hepatic GCK in patients with T2D, confirming the negative correlation between the GCK and FPG [160]. In addition to the previously highlighted variants, another SNP rs13306393 has been observed at a higher frequency in patients with T2D within a Chinese population [161]. Interestingly, this SNP is located at the intron near the liver-specific promoter (exon 1b). Therefore, it is exclusively expressed in the liver GCK. Evaluation of such variant showed that the increased risk of T2D is due to insulin resistance rather than impairments in the islet β cell biology [162]. This was the first study to show that GCK variants increase insulin resistance. The association between T2D and SNPs, including GCK rs1799884 G>A, MIR-196A-2 rs11614913 C>T, and MIR-423 rs6505162, have been investigated in a Saudi population. The study revealed a significant association of the AA genotype and A allele of GCK rs1799884 G>A with T2D susceptibility, alongside an association of the MIR-196A-2 rs11614913 CT genotype and T allele, as well as the MIR-423 rs6505162 CA genotype, with T2D [163].

Overall, these findings underscore the impact of *GCK* variants on T2D phenotypes and highlight the importance of genetic factors in disease susceptibility and treatment response. Additionally, they provide valuable insights for future research endeavors aimed at elucidating the intricate genetic architecture of diabetes and enabling personalized therapeutic strategies.

## Modeling GCK-associated monogenic diabetes: insights from animal and cell models

In light of GCK’s involvement in various forms of MD, animal models have been established to study the role of GCK in developing hyperglycemia and hypoglycemia (Table 2) [96, 117, 155, 164]. Early studies attempted to generate homozygous global Gck knockout (KO) mice to mimic the phenotypes of PNDM [96, 164]. However, these studies were unsuccessful in producing mice that completely lack Gck, as its absence has been shown to be lethal during development [96, 164].

**Table 2:** *Gck* knockout mouse models

KO models	Clinical phenotypes	References
*Gck* KO mouse models	Embryos die at day 9.5	[96]
	Mice develop hyperglycemia after the second day of birth and die within the first week	[155, 164]
	Mouse survive for 5 weeks after birth	[155]
KO model of pancreatic *GCK*	Die a few days after birth due to severe diabetes	[117]
KO model of hepatic *GCK*	Mild hyperglycemia with defect in the synthesis of glycogen	[117]
Heterozygous deficiency of global or pancreatic *GCK*	Moderate hyperglycemia	[117, 164]
Heterozygous deficiency of hepatic *GCK*	In the liver, decrease in the mRNA level of insulin receptor and Glut2 expression In the pancreas, there is a large amount of glucagon secreted from α-cells In muscles, hexokinase II (HKII) is decreased while there is no obvious change in adipocyte tissue	[52]

Nevertheless, some studies employed *N*-ethyl-*N*-nitrosourea (ENU)-induced mutagenesis strategy to generate Gck mutant mice through the induction of specific point mutations [155, 156]. These studies successfully generated mice with homozygous GCK mutations [155, 156]. The use of ENU-induced mutations revealed that different types of homozygous GCK mutations result in different phenotypes. For example, mice with a splicing donor mutation in the β-cell exon 1 (M-210) suffer marked hyperglycemia and severe growth retardation, leading to death within the first week after birth [155]. In contrast, mice with the missense mutation Va1182Met (M-392) survive up to 5 weeks after birth despite having a functionally impaired GCK protein [155]. Taking all together, these findings highlight the importance of GCK during development and underscore that the severity of phenotype depends on the type of homozygous *GCK* mutation.

To elucidate the role of GCK in specific tissues, β-cell and liver-specific *Gck* KO models have been generated through Cre-LoxP gene-targeting technology [165]. Mice lacking β-cell specific Gck exhibit severe hyperglycemia and die shortly after birth [165]. This β-cell-specific *Gck* KO mouse model is similar to mutant mice generated by Terauchi et al., which lack the expression of the neuroendocrine isoform of Gck in all sites, including islets, brain, and gut [166]. The similarity between these two distinct models indicates that the phenotype observed in the neuroendocrine isoform *Gck* KO is mainly due to the loss of β-cell specific GCK, highlighting its essential role in glucose homeostasis [165, 166].

In contrast to β-cell specific *Gck* KO, liver-specific *Gck* KO mice survived. However, in vivo, analysis of mice with liver-specific *Gck* KO showed marked impairment in glycogen synthesis and unexpectedly in insulin secretion [165]. To further characterize the role of liver-specific GCK in glucose homeostasis, Zhang and his colleagues generated a mouse model that lacked liver-specific GCK. The later mouse model showed a decrease in the levels of Gck protein and activity with age [159]. At the age of 6 weeks, impaired glucose tolerance and elevated fasting glucose levels are observed [159]. Importantly, this phenotype is independent of the pancreatic β-cells, as the function of β-cell Gck is normal in mice at a young age [159]. These results highlight the importance of liver GCK in maintaining glucose homeostasis and present an ideal animal model to study the pathogenesis of MODY2 [159].

In addition to homozygous Gck KO mice, global and β-cell-specific heterozygous Gck mutant mice have been generated. Both types of heterozygous Gck mutants showed a hyperglycemic phenotype and a defective insulin secretion in response to glucose. In vivo analysis of mice with global heterozygous Gck mutation showed a marked glucose intolerance during hyperglycemic clamp studies [165]. This glucose intolerance phenotype was similar to a previously reported mouse model generated by Bali et al., which also showed glucose intolerance in adult mice with a single functional GCK gene copy [96, 165]. Importantly, these studies confirmed that GCK haploinsufficiency impaired insulin secretion and provided a relevant model for MODY2.

Different research groups have also developed transgenic mice overexpressing the *Gck* gene. Several studies showed that mice with increased expression of pancreatic and liver-specific Gck showed an increase in hepatic glucose metabolism and a reduction in the levels of plasma glucose levels [160, 167]. Similar findings were observed following the overexpression of GCK in the livers of both fasted and fed rats, as well as in diabetic mouse models. Collectively, these studies displaying increased liver Gck expression and reduced plasma glucose levels, suggested a promising therapeutic avenue for diabetes treatment following GCK overexpression [162, 163]. However, they also observed adverse effects of Gck overexpression, such as altered lipid metabolism with elevated serum triglyceride levels [162]. To assess the long-term impacts of hepatic Gck overexpression, Ferre et al. produced transgenic mice overexpressing Gck in the liver through the PEPCK promoter at 12 months of age [168]. Their findings indicate that prolonged Gck overexpression leads to increased hepatic lipogenesis and circulating lipid levels, potentially contributing to an insulin-resistant phenotype and diabetes onset in these mice [168]. These results, along with previous reports, raise concerns regarding GCK manipulation due to its adverse effects on lipid metabolism.

Recently, Chen et al. generated a unique mouse model, as it manipulated the Gck activity in a small population of β-cells through the use of the αGSU-Cre transgene, which is active in a small subset of islet β-cells [169]. To induce Gck deletion in a subset of β-cells, the αGSU-Cre mouse has been crossed with Gck floxed conditional mouse, previously characterized by Postic et al. [165, 169]. Conversely, to activate Gck in a small subset of β-cells, the αGSU-Cre mouse has been crossed with GCK mutant mouse harboring ins454A activating mutation [169, 170]. Characterization of the generated mice showed that genetically activated GCK in a subset of β-cells is efficient in changing the glucose threshold for insulin secretion, and hence, the glucose homeostasis in the whole animal [169]. On the contrary, GCK inactivation in a minority of β-cells has no effect on glucose homeostasis. These results suggest that increased activity of GCK in a minority of β-cells directs them to function as a regulatory trigger for insulin secretion across the entire islet structure [169].

**Fig. 5:**
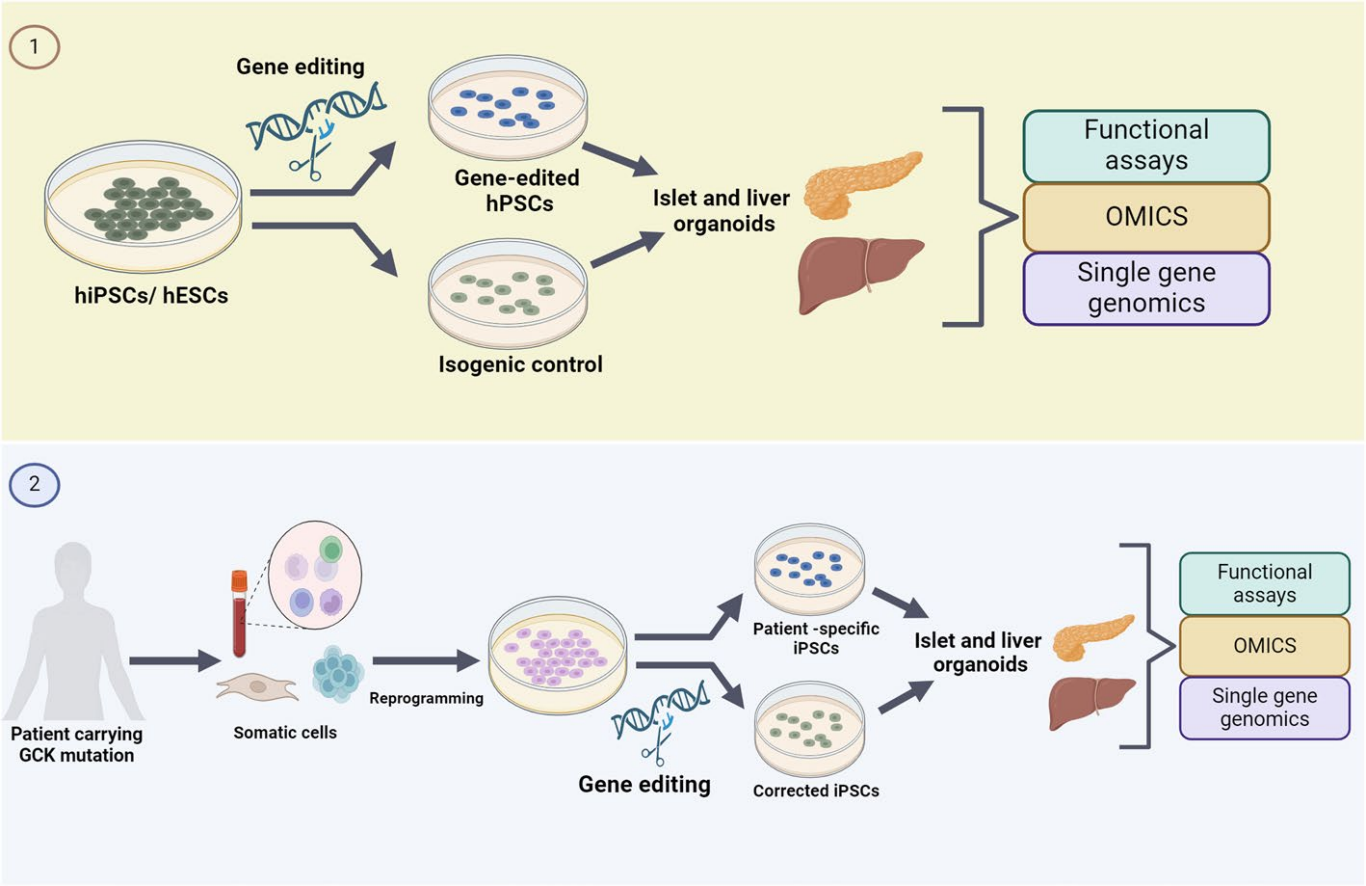
Schematic representation of the hPSC-based approaches for modeling monogenic diabetes caused by *GCK* mutations. Modeling monogenic diabetes (MD) caused by *GCK* mutations can be achieved by utilizing human pluripotent stem cells (hPSCs). One approach entails introducing *GCK* mutations or knocking out the *GCK* gene using gene editing tools on preexisting hPSC lines (1). Alternatively, induced pluripotent stem cells (iPSCs) can be generated from patients with *GCK* mutations (patient-iPSCs), followed by correcting the mutation using genome editing tools (2). These hPSC lines can then be differentiated into β-cells and liver cells to study the impact of the mutation or edited gene on the development and function of the β-cells and liver cells

Overall, the use of animal models has contributed significantly to understanding the role of GCK in glucose homeostasis. However, it is important to acknowledge that these models do not entirely mimic all human pancreas development and diabetes pathogenesis due to inherent variances in physiology and metabolism [53, 171, 172]. Therefore, there is a need for robust preclinical models to study the molecular mechanisms of GCK mutations/variants. Leveraging advancements in hPSC technology, researchers have strived to develop more reliable models. These hPSCs, including human embryonic stem cells (hESCs) and human-induced PSC (hiPSCs) can differentiate into all cell types. Importantly, hPSCs generate patient-specific cells, thereby recapitulating the genetic signature of the patients with diabetes (reviewed in [173–175]). Moreover, stem-cell-based models have demonstrated their efficacy in serving as a powerful tool for modeling CHI [176]. More recently, multiple hiPSC lines have been successfully generated from patients with MODY2 and PNDM due to heterozygous and homozygous mutations in the *GCK* gene, respectively (c.437 T>C) [150]. These models offer promising avenues for studying GCK mutations and developing targeted therapies (Fig. 5).

## Conclusions and future perspectives

The pivotal role of GCK in glucose metabolism, particularly in pancreatic islets and the liver, underscores its significance in maintaining blood glucose homeostasis. Structural and functional insights into GCK have elucidated its unique characteristics and regulatory mechanisms, providing a foundation for understanding its contribution to diabetes pathogenesis. Mutations and variants in the *GCK* gene are associated with various monogenic and polygenic forms of diabetes, emphasizing the need for robust preclinical models to study disease mechanisms accurately. Animal models have offered valuable insights into GCK-related diabetes, but limitations remain in fully replicating human phenotypes. Recent advancements in hPSC technology show promise in overcoming these challenges, offering patient-specific iPSC and gene-edited hPSC models to unravel the molecular underpinnings of GCK-linked diseases. Moving forward, integrating insights from structural biology, genetic studies, and disease modeling will deepen our understanding of GCK-associated diabetes and pave the way for targeted therapeutic interventions.

## Data Availability

Not applicable.

## Abbreviations

G6P: Glucose-6-phosphate
GCK-HI: Glucokinase hyperinsulinism
GCK: Glucokinase
GKRP: GCK regulatory protein
GSIS: Glucose-stimulated insulin secretion
HH: Hyperinsulinemic hypoglycemia
hPSCs: Human pluripotent stem cells
iPSCs: Induced pluripotent stem cells
MD: Monogenic diabetes
MODY2: Maturity onset diabetes of the young 2
NOS: Nitric oxide synthase
PNDM: Permanent neonatal diabetes mellitus
T2D: Type 2 diabetes
UPS: Ubiquitin–proteasome system
